# Nutrition Therapy by Nutrition Support Team: A Comparison of Multi-Chamber Bag and Customized Parenteral Nutrition in Hospitalized Patients

**DOI:** 10.3390/nu15112531

**Published:** 2023-05-29

**Authors:** Seunghyun Cheon, Sang-Hyeon Oh, Jung-Tae Kim, Han-Gon Choi, Hyojung Park, Jee-Eun Chung

**Affiliations:** 1 College of Pharmacy and Institute of Pharmaceutical Science and Technology, Hanyang University, Ansan-si 15588, Gyeonggi-do, Republic of Korea; cshh1005@hanyang.ac.kr (S.C.); dprtmvkdlvm@naver.com (S.-H.O.); hangon@hanyang.ac.kr (H.-G.C.); 2 Department of Pharmacy, Kyung Hee University Hospital at Gangdong, Seoul 05278, Republic of Korea; jtkim@khnmc.or.kr; 3 Department of Pharmaceutical Services, Samsung Medical Center, Seoul 06351, Republic of Korea; 4 School of Pharmacy, Sungkyunkwan University, Suwon-si 16419, Gyeonggi-do, Republic of Korea

**Keywords:** nutrition support team (NST), parenteral nutrition (PN), claim data

## Abstract

This study aimed to investigate the activity of a nutrition support team (NST) and the trends of multi-chamber bag (MCB) and customized parenteral nutrition (PN) with NST consultations in South Korea. Data were obtained from the National Inpatient Sample Cohort between 2015 and 2020. Three datasets were constructed for NST consultation, MCB-PN product prescriptions, and aseptic preparation of total PN. The intersections of the NST consultation and each PN dataset were compiled into MCB-PN with NST or customized PN with a NST sub-dataset, respectively. Using personal identifiers, the patients’ characteristics were evaluated in the NST cohort. A total of 91,384 reimbursements and 70,665 patients were included. The NST activity had increased by more than 50% over 6 years. Approximately 70% and 11%, respectively, of the NST cohort were classified into two subgroups: MCB-PN with NST (M-NST) and customized PN with NST (C-NST). M-NST had many elderly patients with cancer and showed a higher in-hospital mortality than C-NST (12.6% vs. 9.5%). C-NST included a larger number of patients under the age of 5 years, and the hospitalization period was more extended than M-NST (26.2 vs. 21.2 days). The present study showed that NST activities and the proportion of PN with NST consultation are gradually increasing in South Korea.

## 1. Introduction

Many patients experience malnutrition owing to anorexia, absorption disorders, gastrointestinal (GI) surgery, and GI adverse effects caused by medical treatments [1,2]. Even if patients are currently well-nourished, prolonged treatment could lead to depression, lethargy, and poor quality of life, causing a deterioration in nutritional condition, and vice versa [3]. Improving the patient’s nutritional status has a positive effect on clinical outcomes, such as a shortening of hospitalized days and reduction of mortality rate [4,5,6]. Although the importance of nutrition therapy is continuously reported, the global proportion of malnutrition among hospitalized patients is approximately 40%, even when limiting assessments to the United States and Europe, this proportion exceeds 30% [7].

With this prevalence of malnourished patients, some studies have reported that introduction of a nutrition support team (NST) can reduce the severity of malnutrition and the incidence of related adverse effects [8,9,10]. Accordingly, the necessity of a multidisciplinary NST has been emphasized to provide more precise and safe treatment [11,12]. Responding to the demands for NSTs, the Ministry of Health and Welfare has launched legislation for reimbursement of NST activities in South Korea. The NST necessarily comprises a physician, a nurse, a pharmacist, and an experienced dietitian [13]. Furthermore, research on nutrition support through NST activities has recently been conducted to improve practice quality [14,15,16].

The NST is responsible for evaluating the nutritional status of patients and for screening patients in need of parenteral nutrition [17,18]. PN is generally considered for patients who have difficulty in being provided adequate nutrition through oral and enteral feeding, such as patients at risk for aspiration pneumonia, patients with GI problems [19], patients requiring large amounts of calories [20], and premature infants [21,22]. These patients are often hospitalized for a long time with severe cases, and have a risk of infection and thrombosis [23]. Therefore, PN patients should be intensively managed by a NST. Another important task of a NST is to determine which multi-chamber bag PN (MCB-PN) products are appropriate or whether customized PN should be prepared [24].

Although NST activities are gradually expanding worldwide, most existing studies on NST activities are small- or medium-sized cohort studies (less than 1000 patients) conducted in the United States or Europe [25,26]. Even a study focusing on the utilization of PN with a NST has yet to be reported. Therefore, the present study aimed to investigate the status of NST activities nationwide and to evaluate the trends of NST activities for PN patients requiring intensive nutritional intervention, using claims data of South Korea.

## 2. Materials and Methods

### 2.1. Data Source

This retrospective nationwide cohort study used the Health Insurance Review and Assessment Service (HIRA) database. It is a data source based on the National Health Insurance claims data, representing 98% of the South Korean population [27]. The HIRA reviews the information of health insurance subscribers, such as age, gender, treatment, primary diagnosis, secondary diagnosis, and healthcare provider based on encrypted personal identification codes and reimbursement codes [28]. National Inpatient Sample (NIS) data from 2015 to 2020 were used to assess the NST activity and PN utilization trend because NST consultation fees were enacted in August 2014. The NIS data were a stratified proportional sample of about 1,000,000 inpatients per year for 2015–2016 (sampling ratio, 13%) and 750,000 inpatients per year for 2017–2020 (sampling ratio, 10%).

### 2.2. Dataset and Cohort Construction

**Figure 1:**
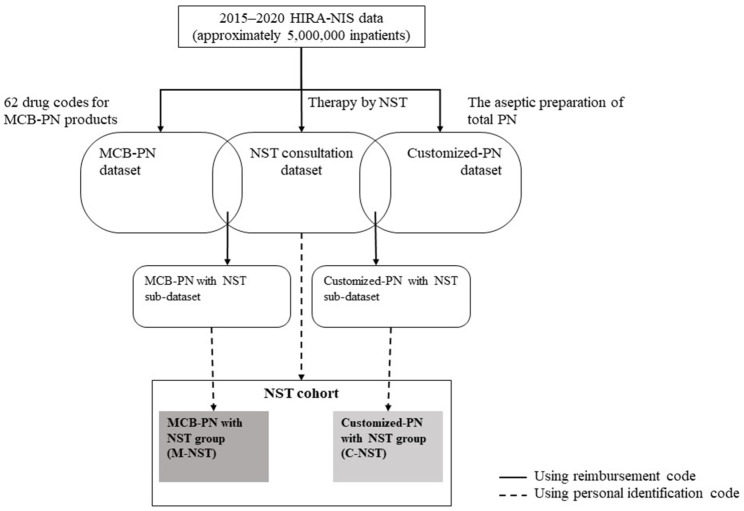
Datasets and cohort construction processes.

The dataset and cohort construction processes of the present study are shown in Figure 1. Three datasets were established using reimbursement codes. First, a NST consultation dataset in which reimbursements claimed insurance for “therapy by nutrition support team” was constructed. There were two categories to indicate whether the healthcare provider of nutrition therapy was a tertiary or general hospital. Second, reimbursements for MCB-PN products were extracted using 62 codes for products distributed in South Korea during study periods. Third, a customized-PN dataset was created using reimbursements for “aseptic preparation of total PN”. Then, reimbursements included in both the NST consultation dataset and MCB-PN dataset were identified and compiled into the “MCB-PN with NST” sub-dataset. Similarly, among the reimbursements of the NST consultation dataset, those also included in the customized-PN dataset were classified as the “customized-PN with NST” sub-dataset.

To confirm the patient information, a NST cohort was constructed using personal identification codes, and two subgroups were also established. Patients who received MCB-PN and NST consultation were classified as the MCB-PN with NST group (M-NST), and patients prescribed customized PN with NST consultation were assigned to the customized PN with NST group (C-NST).

### 2.3. Study Variables and Analysis

To describe the utilization trends in PN therapy with NST, the number of reimbursements and patients were expressed per 1000 inpatients by considering the sampling ratio. Basically, the cohort constructed by using personal identification codes was used to identify the number of patients included in the present study, their gender, their age, and their primary diagnosis based on the International Statistical Classification of Diseases and Related Health Problems 10th Revision (ICD-10). A further analysis was conducted using the reimbursement dataset to analyze the clinical characteristics that may vary by hospitalization when nutritional treatment occurs multiple times in a single year. These characteristics included the medical department, admission route, days of care, days of hospitalization, and healthcare provider’s information. All data were extracted and pooled using SAS^®^ 9.4 version (SAS Institute Inc., Cary, NC, USA). For descriptive statistics, we used percentage or mean (standard deviation). The χ_2_ test or Fisher’s exact test was applied to compare categorical variables between groups, while the Mann–Whitney test was used to compare continuous variables between groups [29].

## 3. Results

### 3.1. NST Consultation Dataset and NST Cohort

A total of 91,384 reimbursements were identified for the NST consultation dataset, and a total of 70,665 patients were included in the present study. The numbers of reimbursements and patients per 1000 inpatients increased by 55.6% and 51.6% over 6 years, respectively. The respective averages were 18.28 reimbursements and 14.13 patients per 1000 inpatients, indicating an average of 1.3 nutrition therapy with NST per patient. The demographic information and the primary diagnosis of these patients are shown in Table 1 and Table 2. The number of patients with NST consultation tended to increase as age increased except in the group aged 0–4 years, and more than 50% of consultations were with elderly patients aged 65 years or older. Moreover, almost half of the nutrition therapies with NST consultation were delivered in an internal medicine (IM) department. When analyzing the IM department in detail, 72.9% were provided in the pulmonology (26.4% of IM and 13.0% of the total), gastroenterology (25.9% of IM and 12.8% of the total), or hemato-oncology (20.6% of IM and 10.1% of the total) department. Following the IM department, the medical departments that frequently prescribed nutrition therapy with NST consultation were general surgery (13.8%), neurosurgery (9.8%), and pediatrics (8.8%). The most common diagnosis was neoplasms (C00-D48) at 27.1%, followed by diseases of the circulatory system (I00-99, 16.0%), diseases of the digestive system (K00-93, 11.6%), and diseases of the respiratory system (J00-99, 10.8%). The most common primary diagnoses were a cerebral infarction (I63), pneumonia (J18), and a malignant neoplasm of the stomach (C16) in that order. The distribution of the primary diagnosis was almost the same in tertiary and general hospitals. Regarding the admission route, 55.6% of patients were hospitalized through the emergency room, 40.6% were hospitalized after visiting outpatient services, and others were hospitalized via transfer from other medical institutions. The average number of hospitalized days was 20.8 days, and the average number of days of care was 28.7 days. The in-hospital mortality rate was 10.3%.

### 3.2. MCB-PN Dataset and M-NST

**Figure 2:**
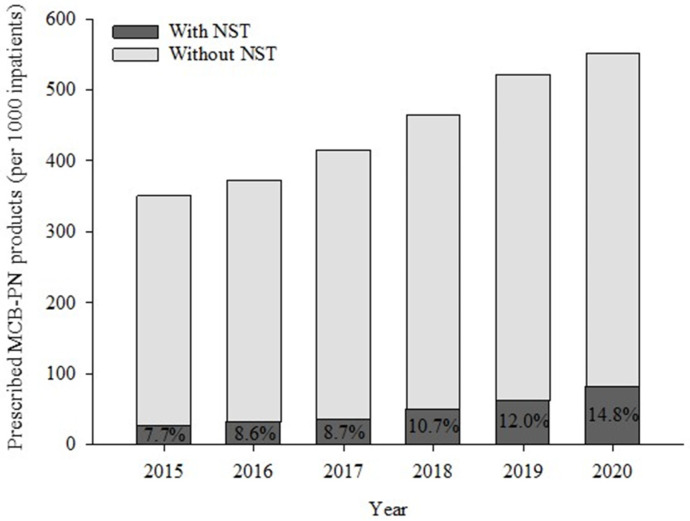
The amount of MCB-PN products prescribed during study periods.

The total number of reimbursement payments for MCB-PN products was 2,188,941 and each reimbursement charged for an average of three products. Over 6 study years, 437.8 products were used per 1000 inpatients. When assessed by year, 350.9 products per 1000 inpatients were used in 2015, and 551.5 products per 1000 inpatients were used in 2020, indicating an increased usage rate over time (Figure 2). Then, the MCB-PN with the NST sub-dataset charging for both MCB-PN and NST consultation comprised 60,916 reimbursements (66.7% of all NST consultation reimbursements). A total of 204,223 MCB-PN products were covered in this sub-dataset over 6 years and they corresponded to 9.3% of the total amount of MCB-PN products prescribed to inpatients. The annual use of NST services is shown in Figure 2, and the proportion of MCB-PN products with NST consultation increased every year from 7.7% in 2015 to 14.8% in 2020. The number of MCB-PN products with NST consultation per 1000 inpatients every year increased from 27.05 in 2015 to 81.47 in 2020. Notably, this usage rate increased by nearly double compared to that of all the prescribed MCB-PN products during the study period (3.01-fold vs. 1.57-fold).

The M-NST comprised 50,010 patients (70.8% of the patients who received nutrition therapy with NST consultation). Over 6 years, an average of 10.00 patients per 1000 inpatients had been prescribed MCB-PN with NST consultation. The increase rate in these patients from 2015 to 2020 was 58.1%, which was greater than that of the NST cohort (51.6%). As shown in Table 1 and Table 2, the overall distribution of the age, gender, medical department, hospitalization route, and distribution of disease organs in M-NST was similar to that in the NST cohort. For patients aged 15 years or older, 76.1% of those with NST consultation were prescribed MCB-PN, and those aged 25–29 years comprised the majority at 81.3%. Among patients aged 0–14 years, only 10.9% of the NST cohort received MCB-PN, including only 5.2% in the 0–4 year age group. With regard to age distribution, the differences from the NST cohort were observed in the proportion of patients aged 0–4 years (0.5% vs. 7.1%), the proportion of pediatrics in the medical department (1.6% vs. 8.8%), and the proportion of certain conditions originating in the perinatal period (P00-96) in the primary diagnosis classification based on ICD-10 codes (0.1% vs. 4.4%). Additionally, there was a significant difference in clinical outcomes. In-hospital mortality in M-NST was higher than in the NST cohort (12.6% vs. 10.3%).

### 3.3. Customized-PN Dataset and C-NST

**Figure 3:**
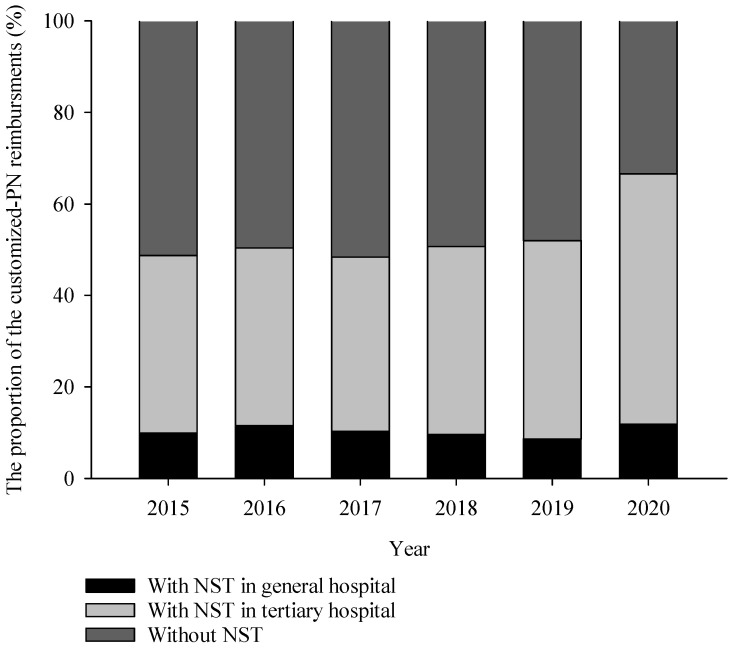
The proportion of customized PN performed with or without NST.

The 17,927 records of insurance reimbursements for “aseptic preparation of total parenteral nutrition” were extracted in the study period, meaning that 3.59 customized PN per 1000 inpatients were administered over 6 years. Among these reimbursements, there were 9343 records of reimbursements that claimed insurance for “therapy by NST” during the same period, which was 52.1% in total. Figure 3 shows the change in ratio of customized PN with or without NST consultation each year. The ratio of customized PN with NST consultation increased from 48.7% in 2015 to 66.6% in 2020. When classified according to the healthcare provider, 80.3% of NST consultations for customized PN were conducted in tertiary hospitals, and the remaining 19.7% were carried out in general hospitals. Furthermore, the proportion of customized PN with NST consultation delivered in general hospitals remained at an average of 10.3% without a certain incremental tendency, whereas the ratio in tertiary hospitals increased from 38.9% in 2015 to 54.7% in 2020 (Figure 3).

The C-NST comprised a total of 7591 patients, indicating 10.7% of patients who received NST consultations. The number of patients who received customized PN with NST consultation per 1000 inpatients increased by 20.7% from 1.39 in 2015 to 1.68 in 2019 and then decreased by 3.3% from 1.68 in 2019 to 1.62 in 2020, for an overall increase of 16.6% over 6 years. Considering the 51.6% growth rate of NST consultations during the study period, the use of the customized PN with NST consultations increased very slowly. One notable point of demographic information in C-NST was the proportion of patients under 5 years of age. As shown in Table 1, this proportion was greater than 40%, in contrast to 0.5% of M-NST. Meanwhile, the proportion of elderly patients aged 65 years or older was low at 22.4%, and this change in the main age group was reflected in the use of medical departments. Pediatrics showed the highest proportion of patients (42.8%), followed by the IM department at 30.0%. The most frequent primary diagnoses for C-NST were the conditions originating in the perinatal period (P00-96) at 29.8%, followed by neoplasms (C00-D48) at 28.2%.

### 3.4. Subgroup Comparison: M-NST vs. C-NST

An analysis of two subgroups in this study showed that 97.9% of patients in M-NST were adult patients ≥ 20 years of age, while only 55.2% in C-NST were adults. The proportion of patients < 5 years of age in C-NST was very high at 42.3%. Our study showed that use of MCB-PN products with NST consultations was almost equal in tertiary and general hospitals (53.5% vs. 46.5%), while customized-PN products predominantly were delivered in tertiary hospitals (78.9% vs. 21.1%). The C-NST patients had lower in-hospital mortality than M-NST patients (9.5% vs. 12.6%). Moreover, C-NST also had more days of hospitalization (26.2 vs. 21.2 days) and days of care (32.4 vs. 28.5 days).

## 4. Discussion

The findings of the present study showed that nutrition therapy with NST consultation was steadily increased over 6 years and NST activities for PN were gradually being actively conducted around MCB-PN. Further, customized PN with NST consultation was mainly prescribed for neonatal patients aged 0–4 years in tertiary hospitals.

Introduction of multidisciplinary NST has been recommended to facilitate precision nutrition therapy of patients, but it has been difficult to achieve the aim in South Korea owing to a lack of professional human resources. Since the fees for “Therapy by Nutrition support team” were newly established in 2014, professionally trained pharmacists, nurses, dietitians, and physicians focusing on nutrition therapy have increased [16]. It is estimated that such increased production of specialists led to the gradual increase of NST activities over 6 years of this study.

PN is usually provided to patients who are not able to intake sufficient nutrition via oral and enteral routes or are severely malnourished [30,31]. It is known that PN has a greater risk of infectious complications compared to enteral nutrition [32,33]. Recently, it was reported that the supply of large-calorie nutrition is more highly related to infection risk than the PN administration method itself [34,35,36]. Therefore, evaluation of the PN supply amount is clinically important. In this regard, one of the NST activities on PN is to determine the proper MCB-PN products to prescribe or whether customized PN should be compounded depending on the patient’s nutritional requirements. In the present study, the proportions of patients receiving MCB-PN or customized PN were about 87% and 13%, respectively. A retrospective study in Singapore also reported similar results (MCB-PN, 78.5% and customized PN, 21.5%) [37]. Conversely, in another study of PN patients in the United States from 2008 to 2014, only about 20% were prescribed MCB-PN and almost 80% received customized PN [38]. This difference is presumed to have occurred due to the different varieties of available MCB-PN products. The available MCB-PN products before 2015 were only two-chamber products and their volumes were also fixed at only 1 or 2 L [39]. The lack of product diversity has led to increased use of individualized compounding products. The present study analyzed 16 types of two-chamber and 46 types of three-chamber products, and their volume varied from 0.5 to 2.053 L. These various options of MCB-PN products mean that appropriate nutrition supply can be performed to patients without compounding customized-PN products. The difference may also be due to the rarity of home–PN therapy. Indeed, a previous study using US insurance claims data showed that most customized-PN products were outsourced to the compounding center [40]. While they are very actively prescribed in the United States [41,42], almost 100% of customized-PN products are compounded in hospital pharmacies because compounding activities in non-hospital institutions are not permitted in South Korea. Even among hospital pharmacies, they can only be performed in qualified institutions that are equipped with a sterile compounding facility that meets the United States Pharmacopeia general chapter 797 (USP <797> Pharmaceutical compounding-sterile preparations) [43]. Automated compounding devices, sufficient pharmacist personnel, and a budget for purchasing consumables also need to be prepared. It is assumed that the difficulty of meeting these requirements in each hospital pharmacy resulted in the predominant use of MCB-PN rather than customized PN. Evaluating the performance ratio of each PN by the healthcare provider type, more than three-quarters of customized-PN therapy was prescribed in tertiary hospitals. In contrast, only half of MCB-PN therapy was delivered in tertiary hospitals. In a previous European survey study, university and non-university hospitals that responded to implement customized PN showed similar results at 78.1% and 36.4%, respectively [44]. This is because it is easier to prepare facilities, equipment, human resources, and the capital necessary for customized-PN implementation in higher-level hospitals [45].

The analysis of the two subgroups in this study showed that 97.9% of M-NST were adults over 19 years of age, while only 55.2% of C-NST were adults. The proportion of patients under the age of 5 years in C-NST was very high at 42.3%. This was interpreted as MCB-PN prescriptions were mainly for adult patients and customized PN was mostly for infants, including premature newborns. This trend is similar to a previous survey conducted in three European countries (Switzerland, France, and Belgium). Approximately 80% of patients with MCB-PN were adults (Switzerland, 86%; France, 79%; and Belgium, 86%), whereas about half of the patients with customized PN were children [44]. It is well known that customized PN is preferred for premature infants due to their wide variety of nutritional requirements depending on body weight, corrected gestational age, and disease severity [46,47]. In addition, some recent studies have reported that the initial excessive energy supply in premature infants adversely affects neurodevelopment, which may support their need for individualized nutrition treatment [48,49]. The difference in the predominant age group between our two subgroups also affected the clinical outcomes; 58.8% of M-NST were elderly patients aged 65 years or older, and their underlying diseases were often life-threatening conditions such as cancer, stroke, and pneumonia [50]. Even if patients with these diseases are hospitalized due to acute exacerbation, it is preferred to conduct management through regular visits after early discharge to prevent hospital-acquired complications [51]. However, premature infants require continuous hospitalization until they are fully developed. Therefore, it was presumed that the difference in patient demographics between our two subgroups resulted in higher in-hospital mortality in M-NST, and longer hospitalization in C-NST.

In the PN with NST group established by combining M-NST and C-NST, 54.1% of patients were over 65 years of age. This is different from the finding of a study using 2018 NIS claims data in the United States, reporting that 40.2% of patients who received PN were elderly [52]. It was presumed to reflect the trend that NST activities are mainly conducted for patients with high malnutrition risk. It is known that cancers, pneumonia, and strokes, which were evaluated as the main diagnoses of NST consultations in the present study, are associated with malnutrition [53,54]. Considering that these diseases are directly or indirectly related to age, it was natural that NST consultations were more frequent in the elderly population than in other age groups. In addition, several studies have found that malnutrition in patients with these diseases is related to high mortality, suggesting the importance of nutritional intervention [2,55,56].

Implementing individualized nutrition therapy for all patients may be effective in preventing adverse effects including hyperglycemia, PN-associated liver disease, and electrolyte imbalance. Nevertheless, the use of MCB-PN is prioritized in adult patients with low nutritional risk because customized PN requires a higher cost, more pharmacist resources, and longer preparation time than MCB-PN [57,58]. With the increasing demand for PN, the NST should ensure patient safety and maximize treatment efficacy using limited types of MCB-PN products. Pharmacists play a central role in the steps from prescription to administration of PN, and should be specialized to ensure appropriate nutrition therapy [59,60].

The present study has a strength in that it analyzed and described the overall utilization status of PN with NST consultations, whereas previous studies have observed malnutrition in patients with certain diseases or patients in the ICU. It is also the first study to report the national utilization status of NST in an Asian country. This study has some limitations. First, we could not ascertain if the patients had actually received the prescribed PN because of using claims data. Although the claims data for inpatients within the hospital were used, there is a possibility that they were not actually implemented. Additionally, the analysis could not include the use of unclaimed PN products. Second, this study excluded the analysis of enteral nutrition, which is another key aspect of nutrition therapy with NST. Third, it did not separately analyze patients who used both MCB and customized-PN products during a single hospitalization stay.

## 5. Conclusions

The present study shows that the activities of NST are continuously increasing in South Korea. NST consultations for customized PN have been primarily conducted for patients of extreme ages, including premature infants. NST activities for PN have been mainly related to prescribing MCB-PN products. These results suggest that the appropriate selection of MCB-PN products with various compositions and volumes has enabled providing precise nutrition treatment for individual patients. This study is the first nationwide description of PN-prescribing patterns with NST. It is expected to be helpful in preparing efficient PN implementation protocols in countries with limited institutional or human resources. Further research is required to analyze the overall NST activity including enteral nutrition as well as PN.

## Figures and Tables

**Table 1:** The demographic information of NST cohort and two subgroups.

		NST Cohort *n* (%)	Comparative Analysis of Subgroups		
			M-NST *n* (%)	C-NST *n* (%)	*p*-Value
No. of patients		70,665	50,010	7591	-
No. of patients per 1000 inpatients		14.13	10.00	1.52	-
Gender	Male	40,190 (56.9)	28,796 (57.6)	4383 (57.7)	*p* < 0.001
	Female	30,475 (43.1)	21,214 (42.4)	3208 (42.3)	
Age	<5	4995 (7.1)	262 (0.5)	3208 (42.3)	*p* < 0.001
	5–9	390 (0.6)	158 (0.3)	67 (0.9)	
	10–14	367 (0.5)	205 (0.4)	47 (0.6)	
	15–19	590 (0.8)	419 (0.8)	76 (1.0)	
	20–24	636 (0.9)	485 (1.0)	48 (0.6)	
	25–29	635 (0.9)	516 (1.0)	34 (0.4)	
	30–34	965 (1.4)	746 (1.5)	60 (0.8)	
	35–39	1235 (1.7)	955 (1.9)	108 (1.4)	
	40–44	1830 (2.6)	1397 (2.8)	150 (2.0)	
	45–49	3045 (4.3)	2299 (4.6)	222 (2.9)	
	50–54	4228 (6.0)	3270 (6.5)	312 (4.1)	
	55–59	6048 (8.6)	4718 (9.4)	455 (6.0)	
	60–64	6636 (9.4)	5167 (10.3)	547 (7.2)	
	65–69	6945 (9.8)	5337 (10.7)	560 (7.4)	
	70–74	8323 (11.8)	6348 (12.7)	572 (7.5)	
	≥75	23,797 (33.7)	17,728 (35.4)	1125 (14.8)	
No. of reimbursements		91,384	60,916	9343	-
No. of reimbursements per 1000 inpatients		18.28	12.18	1.87	-
Medical department	Internal medicine	44,978 (49.2)	33,395 (54.8)	2807 (30.0)	*p* < 0.001
	Gastroenterology	11,659 (12.8)	9205 (15.1)	812 (8.7)	
	Cardiology	3364 (3.7)	1927 (3.2)	165 (1.8)	
	Pulmonology	11,888 (13.0)	8627 (14.2)	544 (5.8)	
	Nephrology	4175 (4.6)	2765 (4.5)	170 (1.8)	
	Hemato-oncology	9254 (10.1)	7954 (13.1)	868 (9.3)	
	Infection	2439 (2.7)	1619 (2.7)	89 (1.0)	
	Other IMs	2199 (2.4)	1298 (2.1)	159 (1.7)	
	Neuropsychiatry	4851 (5.3)	2462 (4.0)	124 (1.3)	
	General surgery	12,628 (13.8)	10,699 (17.6)	1304 (14.0)	
	Neurosurgery	8998 (9.8)	6114 (10.0)	233 (2.5)	
	Chest surgery	2455 (2.7)	1692 (2.8)	360 (3.9)	
	Pediatrics	8076 (8.8)	991 (1.6)	3997 (42.8)	
	Others	9398 (10.3)	5563 (9.1)	518 (5.5)	
Admission route	Outpatients	37,074 (40.6)	23,477 (38.5)	5086 (54.4)	*p* < 0.001
	Emergency	50,847 (55.6)	34,871 (57.2)	3980 (42.6)	
	Transfer from other institutions	3463 (3.8)	2568 (4.2)	277 (3.0)	
Clinical outcomes	Continuation	26,863 (29.4)	17,163 (28.2)	3553 (38.0)	*p* < 0.001
	Death	9429 (10.3)	7683 (12.6)	891 (9.5)	
	Discharge	49,059 (53.7)	32,411 (53.2)	4507 (48.2)	
	Others	6033 (6.6)	3659 (6.0)	392 (4.2)	
Days of hospitalization (mean ± SD)		20.8 ± 15.0	21.2 ± 15.0	26.2 ± 17.3	*p* < 0.001
Days of care (mean ± SD)		28.7 ± 18.7	28.5 ± 18.0	32.4 ± 20.6	*p* < 0.001

**Table 2:** The primary diagnosis according to ICD-10 classification of NST cohort and two subgroups.

ICD-10 Classification		NST Cohort *n* (%)	M-NST * n (%)	C-NST * n (%)
A00–B99	Certain infectious and parasitic disease	1740 (2.5)	1287 (2.6)	90 (1.2)
C00–D48	Neoplasms	19,149 (27.1)	16,683 (33.4)	2142 (28.2)
D50–D89	Diseases of the blood and blood-forming organs and certain disorders involving the immune mechanism	264 (0.4)	142 (0.3)	14 (0.2)
E00–E90	Endocrine, nutritional, and metabolic diseases	815 (1.2)	426 (0.9)	44 (0.6)
F00–F99	Mental and behavioral disorders	284 (0.4)	178 (0.4)	11 (0.1)
G00–G99	Diseases of the nervous system	1815 (2.6)	960 (1.9)	108 (1.4)
H00–H95	Diseases of the eye and adnexa and diseases of the ear and mastoid process	71 (0.1)	34 (0.1)	3 (0.0)
I00–I99	Diseases of the circulatory system	11,303 (16.0)	7093 (14.2)	433 (5.7)
J00–J99	Diseases of the respiratory system	7667 (10.8)	5604 (11.2)	381 (5.0)
K00–K93	Diseases of the digestive system	8174 (11.6)	6634 (13.3)	612 (8.1)
L00–L99	Diseases of the skin and subcutaneous tissue	268 (0.4)	145 (0.3)	18 (0.2)
M00–M99	Diseases of the musculoskeletal system and connective tissue	958 (1.4)	583 (1.2)	47 (0.6)
N00–N99	Diseases of the genitourinary system	3124 (4.4)	2070 (4.1)	137 (1.8)
O00–O99	Pregnancy, childbirth, and the puerperium	44 (0.1)	26 (0.1)	2 (0.0)
P00–P96	Certain conditions originating in the perinatal period	3144 (4.4)	65 (0.1)	2260 (29.8)
Q00–Q99	Congenital malformations, deformations, and chromosomal abnormalities	587 (0.8)	65 (0.1)	337 (4.4)
R00–R99	Symptoms, signs, and abnormal clinical and laboratory findings, not elsewhere classified	1210 (1.7)	759 (1.5)	73 (1.0)
S00–T98	Injury, poisoning, and certain other consequences of external causes	4416 (6.2)	3089 (6.2)	215 (2.8)
U00–U85	Codes for special purposes	79 (0.1)	63 (0.1)	8 (0.1)
V01–Y98	External causes of morbidity and mortality	0 (0.0)	0 (0.0)	0 (0.0)
Z00–Z99	Factors influencing health status and contact with health services	1365 (1.9)	995 (2.0)	111 (1.5)
Not specified ^#^		4188 (5.9)	3109 (6.2)	545 (7.2)

* means the statistically significant difference between M-NST and C-NST (*p* < 0.001). ^#^ means the inability to identify primary diagnosis due to masking or missing data.

## Data Availability

All data presented in this article are available on request to the corresponding authors.
